# 363. A Phase II Prospective Randomized Study to Assess Imipenem-Relebactam in the Management of Cancer Patients with Febrile Neutropenia

**DOI:** 10.1093/ofid/ofad500.433

**Published:** 2023-11-27

**Authors:** Anne-Marie Chaftari, Hiba Dagher, Ray Y Hachem, Ying Jiang, Peter Lamie, Rita Wilson Dib, Teny John, Andrea Haddad, Ann Philip, Shahnoor Ali, Patricia Mulanovich, Ying Juan, Patrick Chaftari, Issam I Raad

**Affiliations:** MD Anderson UT, Houston, Texas; UT MD Anderson Cancer Center, Houston, Texas; MD Anderson UT, Houston, Texas; The University of Texas MD Anderson Cancer Center, Houston, Texas; UT MD Anderson Cancer Center, Houston, Texas; MD Anderson Cancer Center, Houston, Texas; MD Anderson Cancer Center, Houston, Texas; UT MD Anderson Cancer Center, Houston, Texas; MD Anderson Cancer Center, Houston, Texas; MD Anderson Cancer Center, Houston, Texas; MD Anderson Cancer Center, Houston, Texas; MD Anderson Cancer Center, Houston, Texas; UT MDAnderson Cancer Center, Houston, TX; MD Anderson UT, Houston, Texas

## Abstract

**Background:**

The overuse of antibiotics has led to the emergence of antibiotic resistance that poses a significant threat particularly for the immunocompromised cancer patients. Hence, the empiric therapy with standard antibiotic could be suboptimal in these vulnerable patients who often receive antibiotic treatment. New antibiotics that cover potential resistant pathogens may be considered.

In this prospective study we wanted to evaluate the role of a new combination of β-lactam/β-lactamase inhibitor Imipenem-Relebactam (IR) in comparison to standard of care (SOC) antibiotics in the empiric treatment of cancer patient with febrile neutropenia.

**Methods:**

We conducted a prospective, randomized, open-label study comparing the safety and efficacy of IR vs SOC antibiotics (cefepime, piperacillin/tazobactam or meropenem) used in combination with gram-positive antibacterial agents as empiric therapy in adult cancer patients with febrile neutropenia. Patients received at least 48 hours of intravenous (IV) antibiotics before any switch of antibiotic was allowed and were followed through end of intravenous (EOIV) therapy and for up to 42 days.

**Results:**

A total of 80 patients were enrolled between October 2021 and April 2023 of whom 39 were randomized to IR and 41 to SOC. Age, gender, race, and underlying malignancies were similar in both groups. The rate of microbiological infection including bacteremia was similar in both groups (27% in IR vs. 31% in SOC; p=0.81). Favorable clinical response at EOIV was comparable in both groups (88% in IR vs. 72% in SOC; p=0.12). The microbiological eradication at EOIV was 100% in IR vs 75% in SOC; p=0.22. Study-drug related adverse events were similar in both groups (10% in IR vs. 18% in SOC; p=0.29). Although overall mortality at late follow up was higher in SOC arm (11% in SOC vs. 0 in IR; p=0.049), there was no study-drug related mortality.

**Conclusion:**

The use of IR as empiric coverage of febrile neutropenia in cancer patient is efficacious and safe and could be associated with a lower overall mortality.

**Disclosures:**

**Issam I. Raad, Distinguished Professor**, Novel Anti-Infective Technologies, LLC: Technology License

